# Widowhood, social networks, and mental health among Chinese older adults: The moderating effects of gender

**DOI:** 10.3389/fpsyg.2023.1142036

**Published:** 2023-04-03

**Authors:** Dan Tang, Christine A. Mair, Qing Hu

**Affiliations:** ^1^Center for Population and Development Studies, Institute of Gerontology, Family and Gender Study Center, Renmin University of China, Beijing, China; ^2^Department of Sociology, Anthropology and Public Health, University of Maryland, Baltimore County, Baltimore, MD, United States; ^3^Institute of Gerontology, Renmin University of China, Beijing, China

**Keywords:** widowhood, gender, social networks, depressive symptoms, life satisfaction, older adults, China

## Abstract

**Objectives:**

This study aimed to examine the three-way interaction between widowhood, social ties, and gender and its effects on older adults’ mental health, including depressive symptoms and life satisfaction, in the context of China.

**Methods:**

Participants were 7,601 Chinese older adults. Their social network was divided between family and friendship ties, and their mental health was measured by depressive symptoms and life satisfaction. Linear regression was employed to analyze the associations between widowhood, social networks, and mental health, as well as to explore the moderating effect of gender.

**Results:**

Widowhood is associated with more depressive symptoms, but not with life satisfaction, while family and friendship ties are associated with less depressive symptoms and greater life satisfaction. Furthermore, the lack of family ties is associated with more depressive symptoms for widowed men compared to married older men, while it is associated with lower life satisfaction for widowed women compared to married older women.

**Conclusion:**

Family ties are the most important social support resource for Chinese older adults, especially for the widowed group. The vulnerability of older widowed men who lack family ties in China deserves public concern and attention.

## 1. Introduction

The death of a spouse is one of the most stressful and influential events in older adults’ lives (Chen et al., 2019) and can adversely affect physical and psychological well-being (Jadhav and Weir, 2018). However, widowhood may be less harmful for older adults who have strong social connections to family and friends (Li et al., 2005; Zhang and Li, 2011; DeVries et al., 2014). Furthermore, the experiences of widowhood, social ties, and mental health are contextualized by gender and country context (Guo et al., 2017; Jadhav and Weir, 2018). Compared to men, women experience widowhood at a higher rate, have stronger and more diverse social networks, and may experience mental health differently. In terms of country context, in China, support from children is particularly valued and expected, and widowhood can be an especially vulnerable life event for those with fewer options for support–an experience that may become more common as the one-child generation ages and fertility remains low (Hesketh et al., 2005). However, no study to date has examined the potential three-way relationships between widowhood, family and friendship ties, and gender in terms of their effect on older adults’ mental health in China. To address this gap, we combine multiple conceptual frameworks (the convoy model of social networks, buffering models of social support, dual factor model of mental health) to test associations between widowhood and mental health, and explore if these associations are contextualized by family ties, friendship ties, and gender for older Chinese.

## 2. Theoretical background

### 2.1. Social networks and widowhood

The *convoy model* of social networks emphasizes the process through which close personal ties promote well-being during the course of life (Kahn and Antonucci, 1980; Antonucci and Akiyama, 1987; Antonucci et al., 2014). This framework pays particular attention to transitions in social ties as individuals age, such as the experience of widowhood and the resulting shifts that may occur in the social network of the widowed individual (Fuller et al., 2020). Although convoy model scholars have documented the negative consequences of close social ties when they are under strain (Antonucci et al., 1998), the presence of family and friendship ties is generally associated with better mental health, particularly in widowhood (Kang and Ahn, 2018). Widowed older adults experience at least a temporary uptick in contact from family and friends following the death of a spouse, and these ties are associated with better mental health in widowhood (Ferraro et al., 1984; Utz et al., 2002; Zettel and Rook, 2004; Guiaux et al., 2007; Ha, 2008; Donnelly and Hinterlong, 2010; DeVries et al., 2014).

In terms of family ties, relationships with family members are some of the most common sources of support, providing and facilitating companionship and care (e.g., Lopata, 1978; Utz et al., 2002; Donnelly and Hinterlong, 2010; Reinhardt, 2010). In addition to family, friendship ties are an important resource in the lives of older adults, who tend to become more selective of their ties and maintain their highest quality friendships in older ages (Carstensen, 1995).Studies from some Western countries have shown that friendship interaction is positively related to mental health among older adults; however, family ties are not always beneficial, being dependent on the quality of the relationship (Lee and Shehan, 1989; Chopik, 2017; Mair, 2019; Peng et al., 2022). Previous research has also shown that family ties are more important for older adults’ well-being in non-Western cultures (Alegria et al., 2007; Lee et al., 2018).

Social networks are an important source of social support (Antonucci et al., 2014) and may have stronger positive effects on mental health when individuals are in stressful situations, consistent with the buffering model of social support (Cohen and Wills, 1985). Widowhood is a prime example of a stressful situation experienced by older adults, and research showed that the association between social networks and well-being is stronger among the widowed older adults than among those married (Li et al., 2005; Zhang and Li, 2011); however, studies showed inconsistent results (e.g., Stroebe et al., 1996).

### 2.2. Gender differences

Not only are women more likely to be widowed than men, they cultivate social convoys differently, experience widowhood differently, and may also differ from men in terms of their mental health outcomes (Antonucci et al., 2011; Schwartz and Litwin, 2018; Luo et al., 2021; Li et al., 2022). For example, women are more likely to have diverse social networks of family and friendship ties than men, which is linked to better mental health (Connidis, 2010). Furthermore, women are more likely to act as a family “kinkeeper” by promoting contact between family members and sharing information about them (Rosenthal, 1985; Fuller et al., 2020). For these reasons, older women in multiple country contexts appear to have a social well-being advantage compared to older men.

Social disparities between older men and women may become more pronounced in widowhood, with older widowed men being at greater risk of social isolation and poorer mental health. For example, Jadhav and Weir (2018) found that men may experience depressive symptoms up to a decade following widowhood in the United States. However, additional studies showed that gender gaps in depression during widowhood diminish after accounting for length of illness prior to spouse death, marital quality, financial strain, duration of widowhood, education, and especially, family support (Li et al., 2005; Zhang and Li, 2011; Sasson and Umberson, 2014; Jadhav and Weir, 2018; Yang, 2021).

Taken together, there is ample evidence to suggest that the experiences of widowhood, social ties, and mental health may differ between men and women. Therefore, the three-way relationship between these factors should be examined for gender differences in order to make more useful recommendations on how to promote social interaction to enhance widowed older adults’ mental health.

### 2.3. The dual factor model of mental health

Research has considered depressive symptoms as the major independent indicator of the association between marital status and mental health (e.g., Guo et al., 2017; Jadhav and Weir, 2018). However, based on the dual factor model of mental health (DFM), which conceptualizes mental health as having simultaneous positive and negative mental states (Greenspoon and Saklofske, 2001; Keyes, 2007; Suldo and Shaffer, 2008; Westerhof and Keyes, 2010; Wang et al., 2011), a focus on only poor mental health (e.g., depression, loneliness, and psychological distress; Xu et al., 2020; Gyasi and Phillips, 2020; Yang, 2021) is incomplete without considering positive mental health (e.g., life satisfaction, subjective well-being). Although, many options for negative and positive indicators are found in studies based on DFM theory, depressive symptoms and life satisfaction are the most commonly used variables (Keyes, 2007; Iasiello et al., 2020).

As previously mentioned, many studies demonstrated significant associations between losing a spouse and depressive symptoms. However, the associations between widowhood and positive mental health indicators varied in different researches. For example, while negative associations between widowhood and life satisfaction-related health were found in studies (Liu and Guo, 2008; Li et al., 2016), it was not always the case (Li et al., 2015; Ng et al., 2017).

### 2.4. The contextual case of China

We focused on the unique context of China, which represents a potential clash between culture and demographics. According to the Data of the Fourth Sample Survey of the Living Conditions of China’s Urban and Rural Older Persons, the proportion of older adults aged 60 and over who are widowed is 26.1% (China Scientific Research Center on Aging, 2021), while the number of widowed older adults to grow in China, which is expected to reach 118 million in 2050 (Wang and Ge, 2013). Culturally and socially, China places a very strong emphasis on family ties and responsibility for older parents (filial piety; Fan, 2007; Lin and Pei, 2016; Sun, 2017). However, the strong tradition of a family-based model is becoming more difficult to maintain. The one-child policy set a demographic standard of low fertility that has continued long after its termination. This has created a strain on family networks, with an increasing number of older adults relying on a single child or their child’s spouse to provide care (National Bureau of Statistics of China, 2021). Moreover, the internal migration, including that of labor-age population and older adults, also had a negative impact on the family ties of older adults (Tang and Xie, 2021). The society change in China, contrasted with an intense emphasis on family as the basis of support, has created a vulnerable situation for Chinese older adults in the coming decades.

Perhaps because of the shifting demographics toward lower fertility, as well as a range of other cultural shifts, friendship is becoming a more valued trait for Chinese adults and may be particularly important to be incorporated in studies of widows and widowers’ social networks. According to the World Values Survey, the percent of Chinese adults who reported that friends are “rather” or “very” important in life has increased from 73% in the early 1990s to 92% in the most recent 2019–2020 survey (Inglehart et al., 2020). This pattern has been commonly observed as countries entered into the later stages of economic development and its associated cultural-demographic changes (Mair, 2013). The increasing value placed on friendship may offer additional sources of support for widowed older Chinese adults. The roles of social networks, especially friendship ties, in the association between widowhood and mental health among older adults offer plenty of space for research.

Chinese studies of widowhood, social ties, and mental health revealed patterns similar to those in the United States and Europe–with some exceptions. Social ties–especially family ties–are generally beneficial in improving Chinese widowed older adults’ mental health, including depression (Li et al., 2005) and life satisfaction (Li et al., 2016). In terms of social ties, previous studies found evidence that children help lower depression among Chinese widowed older adults (Li et al., 2005) and that social engagement and activity in general is associated with better life satisfaction (Li et al., 2016). While most studies did not directly examine friendship ties and widowhood, Li et al. (2005) did not find evidence that friendship lowered depression among Chinese widowed individuals. In terms of gender differences in widowhood and mental health, Li et al. (2016) found that associations between widowhood and depression did not differ by gender; however, Ng et al. (2017) established that social ties are more likely to enhance women’s life satisfaction than men’s, regardless of marital status.

While these studies of Chinese widowed older adults are very informative, important gaps can still be found in the literature on widowhood, social networks, and mental health–particularly in China. To the best of our knowledge, no study has examined the three-way relationship between widowhood, social ties, and gender on older adults’ mental health in China. Furthermore, because of the country’s cultural emphasis on family ties, most studies examining widowhood and social support among older adults focused solely on family ties or assessed social support generally without considering the role of friendship ties. Overall, the literature on the social lives of Chinese widows and widowers is not yet well developed or appropriately nuanced, as noted by scholars in their recent work examining this topic (Li et al., 2016; Ng et al., 2017).

### 2.5. Questions and hypotheses

**Figure 1:**
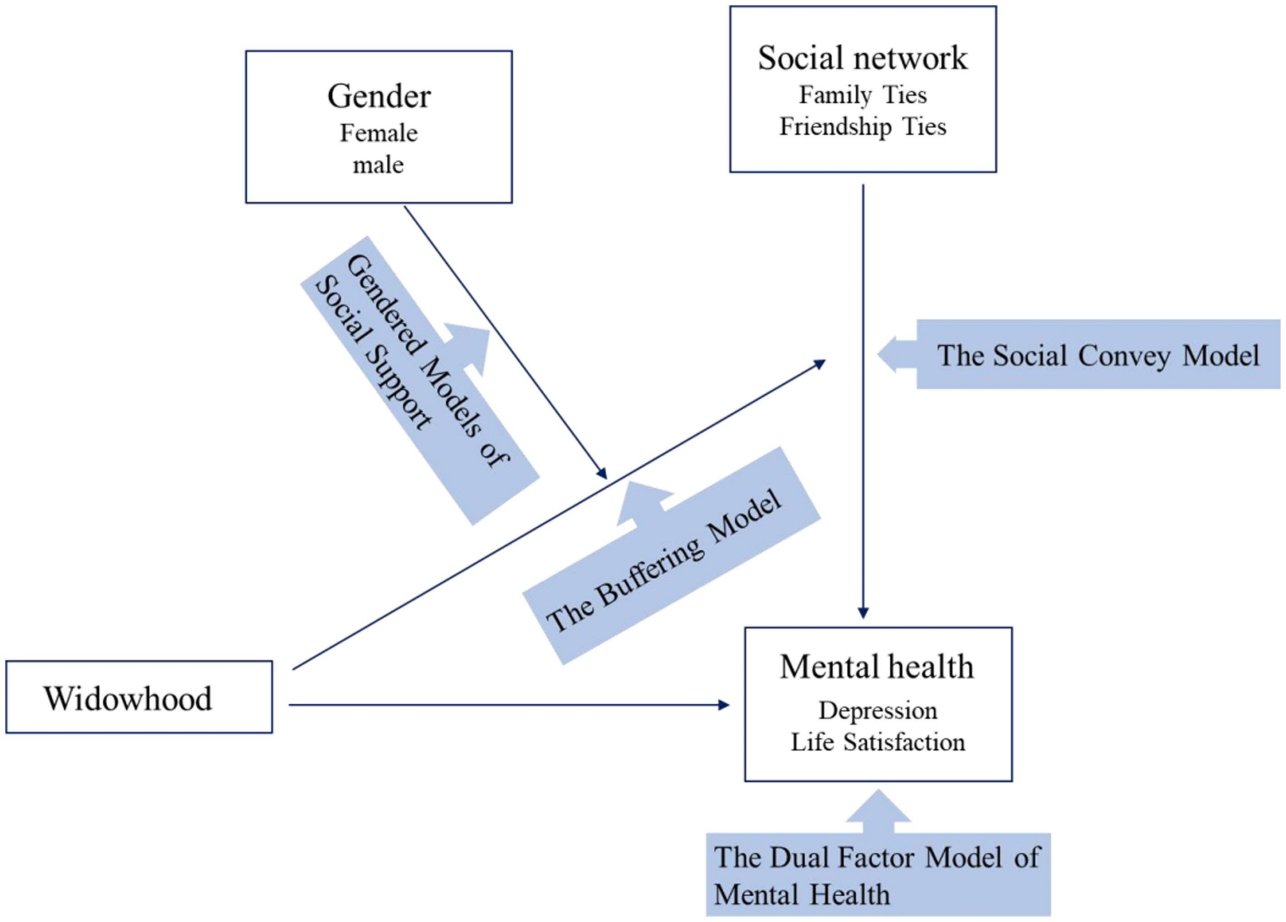
The research framework of the paper.

In this paper, we seek to add to the growing literature on the social lives of Chinese widowed older adults by addressing the gaps described above. Based on the dual factor model of mental health, the first research question is: “Is widowhood associated with depressive symptoms and life satisfaction?” Following the social convoy model, the second research question is: “To what extent are family and friendship ties associated with the mental health of older Chinese?” The third question is based on the buffering model of social support: “To what extent do family and friendship ties moderate (buffer) associations between widowhood and mental health among Chinese older adults?” Last, based on gender difference process, we inquire if the patterns differ by gender. Figure 1 illuminates our research framework.

We anticipate that family and friendship ties are associated with better mental health for older Chinese, and that family ties may have a stronger association with mental health due to the strong emphasis on family ties in the Chinese society. In addition, we expect that family and friendship ties moderate the association between widowhood and mental health, and do so differently by sex, based on gendered models of social support.

To our knowledge, this is the first paper to examine the three-way relationship between widowhood, family and friendship ties, as well as gender on the mental health of Chinese older adults. This gap in the literature is significant and necessary to address in order to document variation and better identify the potential needs of aging widowed men and women in the non-western culture.

## 3. Materials and methods

### 3.1. Data

We used baseline survey data from the China Longitudinal Aging Social Survey (CLASS), conducted by a team of researchers at Renmin University of China^1^ (Tang et al., 2020; Zhao et al., 2020). The baseline survey was conducted between July and December 2014. The CLASS applied a multi-stage stratified probability sampling method, with counties as the primary sampling units, villages/neighborhood committees as the secondary sample units, and people aged 60 and over living in households as the survey respondents. One older adult was randomly selected from each sampled household. The survey covered 28 provinces, autonomous regions, and municipalities in mainland China, and collected information from 11,511 older adults living in 462 villages/neighborhood communities.

Given that the main purpose of this investigation was to examine the role of widowhood in the social networks and depression of older men and women, all respondents who never married, were divorced, or separated were excluded from the sample, to focus on the comparison between those married and widowed (*N* = 11,307). All respondents were initially asked to answer five cognition-related questions and only those who answered correctly on at least three proceeded to the mental health and attitudinal questions (remained *N* = 8,312), consistent with best practices in clinical and epidemiological studies (Wang et al., 2014). Therefore, the findings from this study are not generalizable to the cognitively impaired older population. In addition, we excluded respondents with missing values on the analytical variables (*N* = 711—8.55%; 689 cases missed the variable of income),^2^ resulting in a final sample of 7,601 older adults, including 2,137 widowed (712 male and 1,425 female) and 5,464 married (3,373 male and 2,091 female).

### 3.2. Measures

#### 3.2.1. Dependent variables

The depressive symptoms were measured through a subset (nine items) of the Center for Epidemiologic Studies-Depression (CES-D) scale (Radloff, 1977; Silverstein et al., 2006; Lin and Chen, 2018). Three items indicated feelings of positive affect (feeling happy, enjoying life, feeling pleasure), two items indicated feelings of negative affect (feeling lonely, feeling upset), two items indicated feelings of marginalization (feeling useless, having nothing to do), and two items indicated somatic symptoms (having poor appetite, trouble sleeping). We coded the frequency with which each participant had experienced each symptom in the past week as 0 (*rarely* or *never*), 1 (*sometimes*), or 2 (*most of the time*). After the coding of positive affect items had been reversed, the nine items were summed, producing a depressive symptom score ranging from 0 to 18, with a higher score indicating more depressive symptoms (Cronbach’s alpha = 0.754).

Life satisfaction was measured by one question: “Generally, are you satisfied with your current life?,” which is widely used in measuring life satisfaction cross-nationally, including China (Choi et al., 2016; Han et al., 2020). We coded the answer of the participant as 1 (*very unsatisfied*), 2 (*unsatisfied*), 3 (*modestly satisfied*), 4 (*satisfied*), 5 (*very satisfied*).

#### 3.2.2. Independent variables

Independent variables included widowhood, gender, and social network ties. Widowhood and gender are both dichotomous variables (1 = widowed, 0 = currently married; 1 = male, 0 = female). To assess social network ties, we used the Lubben Social Networks Scale (LSNS; Lubben et al., 2006). LSNS consists of a set of three questions evaluating family ties and three evaluating friendship ties. Questions include: “How many relatives/friends do you see or hear at least once a month?,” “How many relatives/friends do you feel at ease with to talk about private matters?,” and “How many relatives/friends do you feel close to such that you could call on them for help?” For each source of support, we coded the number provided by the participants in their answers for each question as 0 (*none*), 1 (*1*), 2 (*2*), 3(*3 or 4*), 4 (*5 through 8*), or 5 (*9 or more*) and summed these items. This yielded two summation scales ranging from 0 to 15, one for family (Cronbach’s alpha = 0.736) and one for friends (Cronbach’s alpha = 0.846).

#### 3.2.3. Covariates

Covariates included age (in years), living area (1 = urban, 0 = rural), number of surviving children, whether they were living with children (1 = yes, 0 = no), household size (in persons), education level (1 = secondary school or above, 0 = lower than secondary school), annual personal income (in Yuan), functional limitations^3^ (with an index from 0 to 30; higher values indicating more limitations), and number of chronic diseases.^4^ The continuous variables were normally distributed, except for personal income. We used log transformation for personal income in the model estimations; however, the original values are presented in descriptive analyzes.

### 3.3. Analysis

The analysis was conducted in multiple steps. First, we examined the descriptive statistics of all variables for the total sample and conducted bivariate analyzes (*t*-tests) to compare mean scores on all variables (Table 1). Second, we conducted additional bivariate analyzes (*t*-tests) comparing average mental health (depressive symptoms and life satisfaction) and social ties (family and friendship ties) by widowhood status and sex to assess variation by married and widowed men and women (Table 2). Third, we used multivariate linear regression modeling to examine adjusted associations between widowhood, social ties, sex, and two mental health outcomes (depression, Table 3; life satisfaction, Table 4). For each mental health outcome, we included a baseline model of the total sample with all predictors (Model 1) and a two-way interaction model of widowhood*social ties (family and friendship; Model 2). Next, we tested the same two-way interaction model separately by sex (Model 3 for female, Model 4 for male). Taken together, these models examine the interrelationship between widowhood, family and friendship ties, and mental health (depression and life satisfaction) for the total sample as well as for men and women, separately.

**Table 1:** Descriptive statistics of male and female older adults.

	Female	Male	Total	*t*/*χ*^2^ value
	Mean (SD)/%	Mean (SD)/%	Mean (SD)/%	
Depressive symptoms (0–18)	4.84(3.66)	4.35(3.47)	4.58(3.57)	5.907***
Life satisfaction (1–5)	4.11(0.97)	4.08(0.88)	4.10(0.88)	1.655
Widowed	40.5	17.4	28.1	500.306***
Age (60–113)	69.41(7.49)	69.00(7.51)	69.19(7.50)	2.342*
Living in urban area	59.2	55.3	57.1	12.294***
Number of children (0–12)	2.82(1.44)	2.69(1.45)	2.75(1.44)	3.586***
Living with children	46.0	41.5	43.6	15.197***
Household size (1–17)	3.04(1.70)	3.23(1.84)	3.14(1.78)	−4.839***
Secondary education or above	36.1	50.6	43.9	160.152
Annual personal income (in 1000RMB)(0–550 k)	17.86(24.39)	23.30(26.19)	20.79(25.52)	−9.286***
Number of chronic diseases (0–16)	1.83(1.79)	1.53(1.64)	1.67(1.72)	7.752***
Index of functional limitation (0–30)	1.53(3.33)	1.05(2.86)	1.27(3.09)	6.648***
LSNS family ties subscale (0–15)	8.68(3.06)	8.68(3.18)	8.68(3.12)	0.044
LSNS friend ties subscale (0–15)	6.60(4.68)	6.40(4.64)	6.49(4.66)	1.751
*N*	3,516	4,085	7,601	

Values for categorical variables are in percent. The mean values, followed by standard deviations in parentheses, are presented for all other variables.

*Indicates a significant difference (*** *p* < 0.001, ** *p* < 0.01, * *p* < 0.05) between female and male older adults based on chi-square test or *t*-test.

**Table 2:** Lubben Social Networks Scale (Family and Friendship Ties Subscales) and depressive symptoms by marriage in male and female samples.

	Female			Male			Total		
	Married	Widowed	*t*	Married	Widowed	*t*	Married	Widowed	*t*
Depressive	4.33(3.46)	5.58(3.83)	−9.889***	4.10(3.34)	5.57(3.83)	−9.569***	4.19(3.38)	5.58(3.82)	−14.736***
Life satisfaction	4.11 (0.86)	4.12(0.90)	−0.254	4.08(0.88)	4.11(0.87)	−0.783	4.09(0.88)	4.11(0.89)	−1.091
Family Ties	8.77(3.01)	8.55(3.12)	2.043*	8.76(3.15)	8.27(3.27)	3.825***	8.76(3.10)	8.46(3.17)	3.881***
Friendship Ties	6.69(4.68)	6.46(4.67)	1.420	6.53(4.64)	5.79(4.60)	3.894***	6.59(4.65)	6.24(4.66)	3.006**
N	2,091	1,425		3,373	712		5,464	2,137	

*Indicates a significant difference (*** *p* < 0.001, ** *p* < 0.01, * *p* < 0.05) between married and widowed older adults in male, female and total samples, based on *t*-tests.

**Table 3:** Linear regression models predicting depressive symptoms of Chinese older adults: total sample, female and male subsample.

	(1) Total (Baseline)	(2) Total (Interaction)	(3) Female (Interaction)	(4) Male (Interaction)
	B(SE)	B(SE)	B(SE)	B(SE)
Age	−0.035***(0.006)	−0.035***(0.006)	−0.042***(0.009)	−0.030***(0.008)
Urban	−0.447***(0.094)	−0.442***(0.093)	−0.429***(0.140)	−0.374**(0.129)
Number of children	0.110***(0.031)	0.113***(0.031)	0.086(0.047)	0.136***(0.041)
Living with children	−0.320**(0.117)	−0.321**(0.117)	−0.272(0.180)	−0.294(0.155)
Household size	−0.028(0.033)	−0.026(0.033)	−0.109*(0.054)	0.017(0.042)
Secondary education or above	−0.461***(0.086)	−0.457***(0.086)	−0.480**(0.138)	−0.458***(0.111)
Log annual personal income	−0.376***(0.053)	−0.378***(0.053)	−0.280***(0.071)	−0.547***(0.082)
Number of chronic diseases	0.428***(0.022)	0.428***(0.022)	0.435***(0.031)	0.416***(0.030)
Index of functional limitation	0.232***(0.013)	0.232***(0.013)	0.246***(0.018)	0.218***(0.018)
Male	0.082(0.077)	0.081(0.090)	--	--
Widowed	0.945***(0.097)	1.639***(0.255)	1.197**(0.347)	2.107***(0.366)
Family Ties	−0.179***(0.013)	−0.158***(0.015)	−0.164***(0.025)	−0.156***(0.019)
Friendship Ties	−0.047***(0.008)	−0.045***(0.010)	−0.060***(0.016)	−0.034**(0.012)
Widowed*Male		−0.014(0.169)	--	--
Widowed *Family Ties		−0.073*(0.027)	−0.039(0.038)	−0.113**(0.041)
Widowed*Friendship Ties		−0.011(0.018)	0.014(0.025)	−0.045(0.029)
Constant	9.849***(0.425)	9.635***(0.431)	10.165**(0.664)	9.704***(0.580)
*R^2^*	0.222	0.223	0.230	0.214
*N*	7,601	7,601	3,516	4,085

* Indicates the statistical significance (*** *p* < 0.001, ** *p* < 0.01, * *p* < 0.05) in the regression model; B refers to Beta, SE refers to Standard Error.

**Table 4:** Linear regression models predicting life satisfaction of Chinese older adults: total sample, female and male subsample.

	(1) Total (Baseline)	(2) Total (Interaction)	(3) Female (Interaction)	(4) Male (Interaction)
	B(SE)	B(SE)	B(SE)	B(SE)
Age	0.014***(0.002)	0.014***(0.002)	0.013***(0.002)	0.015***(0.002)
Urban	−0.028(0.025)	−0.028(0.025)	0.002(0.037)	−0.059(0.025)
Number of children	0.023**(0.008)	0.013**(0.008)	0.027*(0.012)	0.018(0.011)
Living with children	−0.071*(0.032)	−0.071*(0.032)	−0.145**(0.048)	−0.018(0.043)
Household size	0.007(0.009)	0.008(0.009)	0.041**(0.014)	−0.016(0.012)
Secondary education or above	0.066*(0.023)	0.065(0.023)	0.053(0.036)	0.073*(0.031)
Log annual personal income	0.075***(0.014)	0.076***(0.014)	0.068***(0.019)	0.092***(0.023)
Number of chronic diseases	−0.041***(0.006)	−0.041***(0.006)	−0.041***(0.008)	−0.040***(0.008)
Index of functional limitation	−0.032***(0.003)	−0.033***(0.003)	−0.034***(0.005)	−0.031***(0.005)
Male	−0.082***(0.021)	−0.095***(0.024)		
Widowed	0.003(0.026)	−0.106(0.069)	−0.138(0.091)	0.001(0.101)
Family Ties	0.039***(0.004)	0.029***(0.004)	0.024***(0.007)	0.040***(0.005)
Friendship Ties	0.006**(0.002)	0.009**(0.003)	0.014**(0.004)	0.006(0.003)
Widowed*Male		0.049(0.046)		
Widowed*Family Ties		0.015*(0.007)	0.026**(0.010)	0.007(0.011)
Widowed*Friendship Ties		−0.008(0.005)	−0.012(0.007)	−0.008(0.008)
Constant	2.541***(0.133)	1.838***(0.116)	2.590***(0.202)	2.425***(0.184)
*R^2^*	0.069	0.070	0.073	0.069
*N*	7,601	7,601	3,516	4,085

* Indicates the statistical significance (*** *p* < 0.001, ** *p* < 0.01, * *p* < 0.05) in the regression model; B refers to Beta, SE refers to Standard Error.

## 4. Results

### 4.1. Descriptive and bivariate analysis

Consistent with previous studies, older women in China exhibited higher levels of depressive symptoms than men (Table 1). The widowhood percentage for the total sample was 28.1%, out of which 40.6% belonging to women compared to the 17.4% belonging to men. In terms of social networks, there were no statistically significant differences in family and friendship ties for men and women. Compared to men, women had lower education, lower income, and reported a higher prevalence of chronic diseases.

Compared to married older adults, widowed older adults, both men and women, reported lower scores on family ties and more depressive symptoms (Table 2). Among men, widowed older men reported lower scores on friendship ties compared to married men, while for women, friendship ties did not vary by marital status.

### 4.2. Multivariate regression analysis

**Figure 2:**
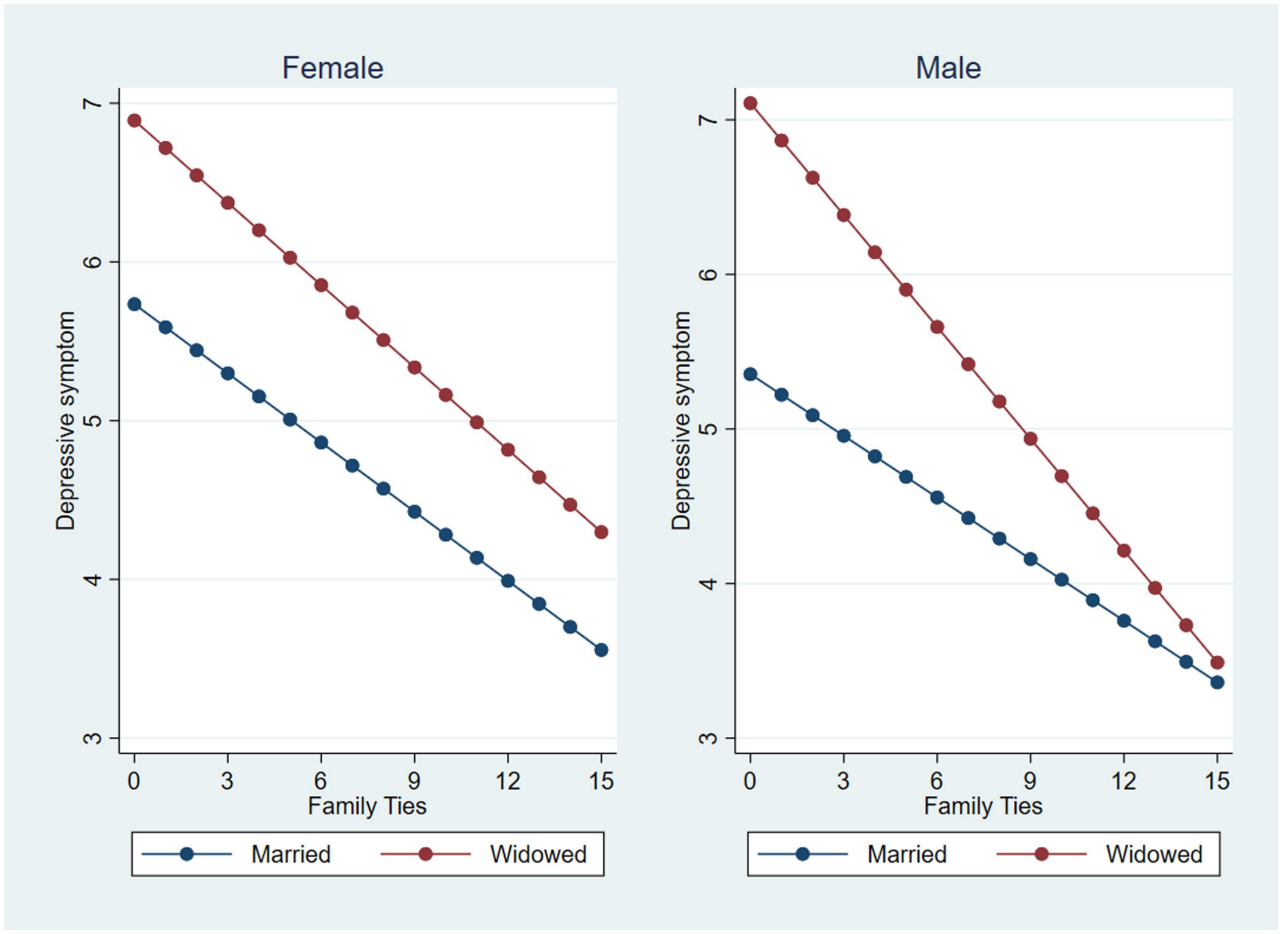
The moderating effect of family ties on the widowhood and depressive symptoms association among female and male older adults.

The first multivariate regression analysis examined predictors of depressive symptoms within the total sample (Table 3). While widowhood was associated with more depressive symptoms (0.945 higher than that of married older adults), the interaction terms between widowhood and gender were not statistically significant. Both family ties and friendship ties (Model 2, Table 3) were associated with a low number of depressive symptoms, with family ties having a stronger association with less depressive symptoms compared to friendship ties (−0.179 vs. -0.047). A test of interaction terms between widowhood and each of the social network ties (family and friends, Model 2) yielded a statistically significant interaction between widowhood and family ties when predicting depression, suggesting that the association between more family ties and less depressive symptoms is stronger for widowed older adults [−0.158 + (−0.073) = −0.231] compared to those married (−0.158). We explored a potential three-way interaction by testing a two-way interaction between widowhood and social network ties in separate models for men and women (Model 3 and 4, Table 3). These models were generally consistent with those of the combined sample in terms of associations between more social network ties (family and friends) and less depressive symptoms. While these patterns were relatively similar for men and women, there was a statistically significant effect of the interaction between widowhood and family ties on depression for the sample of older men only (−0.113), suggesting that family ties may have a stronger association with less depressive symptoms for men compared to women. Figure 2 provides a sex-based visual summary of the associations between widowhood and family ties and its effects on depression. Figure 2 shows that widowers without any family ties are most vulnerable to depressive symptoms.

**Figure 3:**
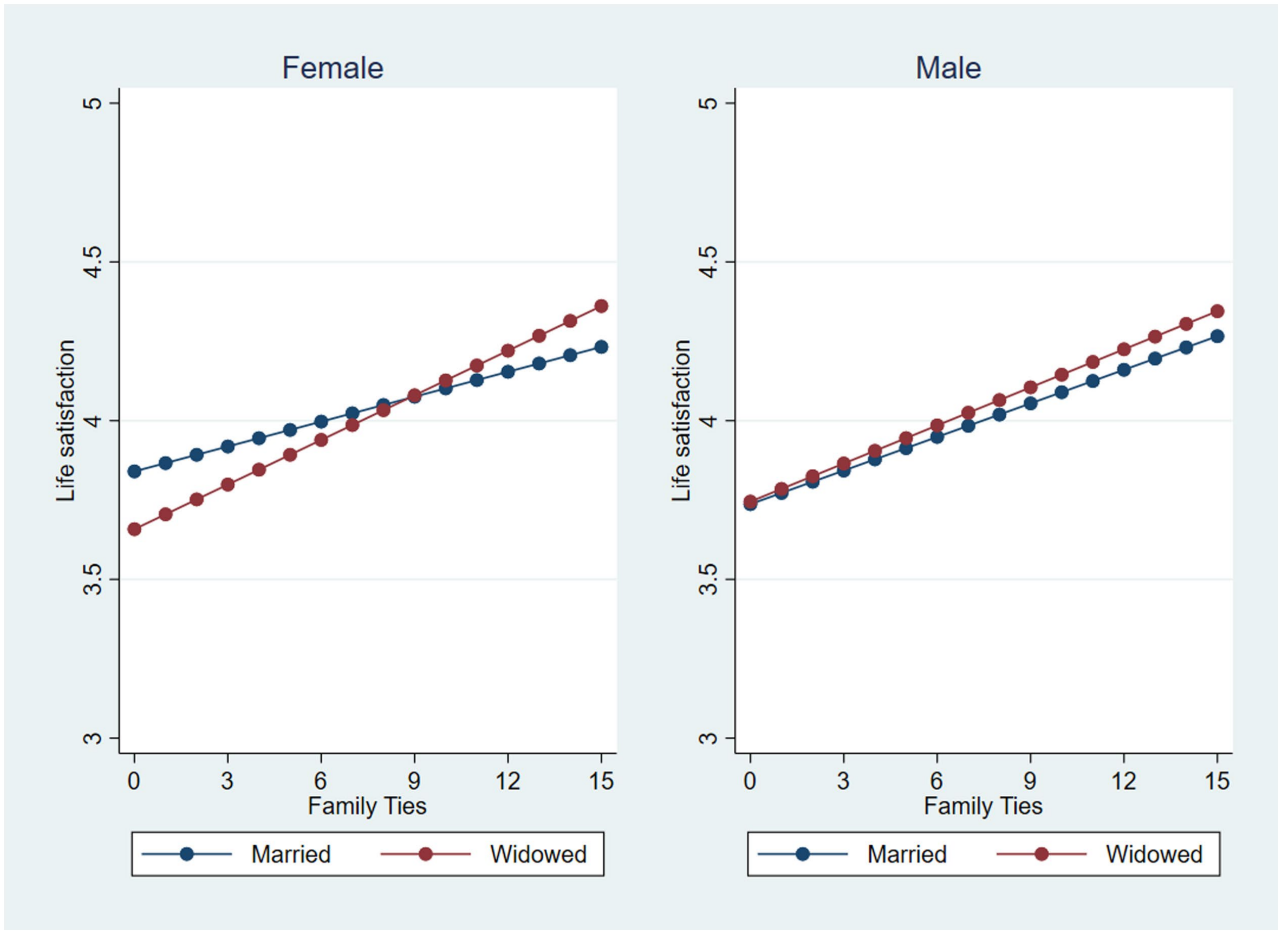
The moderating effect of family ties on the widowhood and life satisfaction association among female and male older adults.

Finally, we repeated the above analyzes with life satisfaction as the dependent variable (Table 4). The results differed somewhat from the regression with depressive symptoms as the dependent variable. Widowhood was not associated with life satisfaction and neither of the interaction terms between widowhood and gender were statistically significant. Both family and friendship ties (Model 2; Table 4) were associated with higher life satisfaction. Similar to depression, family ties had a stronger association with a higher life satisfaction compared to friendship ties (0.039 vs. 0.006). The interaction term between widowhood and family ties was statistically significant (Model 2), suggesting that the association between family ties and better life satisfaction is stronger for widowed (0.029 + 0.015 = 0.44) than married (0.029) older adults. The test of the three-way interaction for life satisfaction (*via* testing a two-way interaction between widowhood and social network ties in separate models for men and women–Model 3 and 4, Table 4) yielded a statistically significant interaction between widowhood and family ties for older women only (0.026). This indicates that the association between family ties and life satisfaction among widowed adults is stronger for women than for men. Figure 3 provides a visual summary of associations between widowhood and family ties on life satisfaction, divided by sex. Figure 3 shows that widows with higher levels of family ties exhibit no differences in life satisfaction compared to married women.

## 5. Discussion

To our knowledge, this paper is the first to examine the three-way interrelationship between widowhood, social ties, and mental health by gender among Chinese older adults. We found evidence that social ties moderate the relationship between widowhood and mental health for older adults in China, and that they do so differently by social tie type, gender, and mental health outcome.

In the total sample, widowhood was associated with more depressive symptoms but not with a lower life satisfaction. Family and friendship ties are both associated with lower depression and higher life satisfaction. When examining these factors jointly, we found evidence that family ties moderate the association between widowhood and mental health for both mental health outcomes, but may do so differently by gender. For widowers, having fewer family ties is associated with higher depression. For widows, having fewer family ties is associated with lower life satisfaction. In other words, having fewer family ties in China places older widowers at risk of poor mental health and reduces positive mental health among older widows. These findings reflect the important role of social ties—especially family–in China, underscore potential differential risk factors for men and women, and raise new questions regarding the future role of friendship for older widows and widowers in the Chinese country context.

### 5.1. The impact on negative emotion of widowhood

Most studies observed significant associations between losing a spouse and depressive symptoms (e.g., Li et al., 2005; Jadhav and Weir, 2018; Wu and Sheng, 2020). Furthermore, while not a constant result, negative associations between widowhood and life satisfaction health were also found (Liu and Guo, 2008; Li et al., 2015, 2016; Ng et al., 2017). This study combined the two aspects of mental health in a single framework and found the distinguishing impact of widowhood on depressive symptoms. Results showed that the loss of a spouse may lead older adults to exhibit an “incomplete mental illness” (Keyes, 2007), meaning that being widowed simply increases the negative emotions of older people, without reducing their positive emotions. More attention needs to be paid to reduce the negative emotions by providing mental and spiritual support, rather than simply meet their life needs through material support.

### 5.2. Family ties particularly important for the widowed in China

In China, similar to other countries, widowhood is a risk factor for poorer mental health. Both older men and women in China report greater depression when widowed. Widowhood continues to be a very disruptive life event for older adults, and deserves constant attention in gerontological work. We found evidence that both family and friendship ties are associated with lower depression and better life satisfaction among those who are widowed and married. In other words, this sample proved that friend and family ties enhance mental health. However, family ties have a much stronger association with both mental health outcomes, underscoring the important role of the family in the context of most countries–including China. When examining the joint relationship between widowhood and social ties, only family ties moderated the association between widowhood and mental health. For widowed Chinese older adults, having more family members available appears to be a particularly important protective factor that reduces depression and enhances life satisfaction. Descriptively, married individuals have more family ties than widowed, placing widowed Chinese at a social disadvantage that deserves consistent scholarly attention.

### 5.3. Men’s increased depression, Women’s decreased satisfaction

The gender differences in the joint association between widowhood, family ties, and mental health make for an informative comparison of the experiences of men and women in widowhood. Women may have greater resilience to widowhood than men. Evidence from Europe indicated that women aged 50 and older were more prone to create new close ties *via* strengthening peripheral ties, while men may prefer more familial and well-known ties, thereby diversifying the potential types of support which are available to elderly female (Schwartz and Litwin, 2018). Although women and widows in this sample reported higher depression on average, it is widowed men that appear to suffer the most when there are fewer family ties. For widowers, lack of family ties may lead to actual sickness (e.g., depression), but for widows that lack of family ties leads them to be dissatisfied but not more depressed. Descriptively, men and women do not differ in their levels of family or friendship social ties (Table 1), and widowed men and women are remarkably similar in their average levels of depression, life satisfaction, and family ties (Table 2). Despite these similarities, the potential effects of family ties vary by gender and mental health outcomes. For widowed women, a lack of family ties is not ideal but is not linked to poor mental health either. Women may be less satisfied, but they are not depressed. The outcomes are different for widowed men, for whom family ties are particularly potent and their lack thereof places them at greater risk of poor mental health. This presents an important possibility for health interventions. Gender Differences in Social Network were also be found in older adults in Australia, it was the same situation in gender difference in association between widowhood and social network that widowhood had a significantly greater negative impact on men’s social networks (McLaughlin et al., 2010).

Indeed, older Chinese widowed men have been previously identified as a population to be targeted in efforts to promote successful aging (Riggs, 1997). National policy efforts in China should continue to support family members and especially children who are supporting parents to enable more frequent visits and contact (Ha, 2010). Older Chinese men of the generation examined in this study may be in particular need of assistance with daily tasks after the loss of their wife, and therefore directing resources to support family members and others who can assist is crucial in maintaining the well-being of this population. Furthermore, national and local policies should continue to promote financial assistance to families, especially when a child is providing support to a widowed parent.

### 5.4. Lack of friendship as additional risk factor for widowed men

Although friendship ties did not moderate the association between widowhood and mental health for Chinese older adults, a few patterns emerged about friendship that may offer insight into additional options for promoting mental health for those who may not have plentiful family ties. The lack of friendship ties was consistently associated with higher depression and lower life satisfaction for Chinese older adults. Moreover, widowed men had the fewest friendship ties of any group in the sample. These patterns suggest additional risk factors for widowed older men in China. Widowed older men have fewer friends, and then suffer particularly when they also have less family.

These results suggest that the friendship patterns of older widowed men in China may be a crucial area for future research. Family ties are not easily added to one’s network and availability of children may be low for some Chinese older adults due to low fertility from the one-child policy. Yet, studies outside of China have demonstrated the benefit of friendship. For example, previous work suggests that the commonality found within friendship peer networks of fellow widowed older adults may be particularly effective in promoting mental health (Morgan et al., 1997), and that friendship ties may be flexible and particularly positive for older widowed older adults especially when family relationships are strained (Morgan, 1989). However, more research is needed on the topic of friendship as a buffer to depression, particularly among older widowed adults in China.

### 5.5. Limitations and future research

Although this work contributes importantly to research on widowhood and mental health in China by identifying various protective factors and risk factors, it has a number of limitations. First, our analyzes are cross-sectional and therefore we cannot make any claims of causality, particularly regarding mental health. Mental health and social networks are deeply intertwined, and those who are experiencing depression are less likely to cultivate ties. Older Chinese widowed men, for example, who are depressed may be less likely to form friendships and maintain relationships with family (reverse causality). Additional research using longitudinal data on the three-way associations between widowhood, social ties, and mental health by gender is warranted. In addition, the sample analyzed in this study includes only community-dwelling older adults with complete cognitive function and therefore caution should be used when extending the results to populations that are institutionalized and/or experiencing cognitive impairment. Finally, we used the Lubben social network scale and two indicators of mental health, but a variety of measurement options exist for each of these concepts that should continue to be explored in the literature.

In conclusion, our results underscore the practical role of social network ties in promoting mental well-being among aging populations, particularly among widowed older adults in China, and highlight some potential pathways for intervention and policy reform. The Chinese context is a unique intersection of cultural norms and national policy promoting family responsibility, yet low rates of fertility and children to support aging parents. Older widowed older adults in China experience higher rates of depression and lower family support, but greater friendship support and a significant mental health boost from their family support networks–particularly older widowed men. These topics will continue to be important for designing policy to support a growing population of Chinese older adults. Our study provides further evidence of the risks and resources potentially available to older widowed older adults in the context of China, but research on social networks and the mental health of older widowed Chinese men and women remains limited and more work is needed to inform policy and practice development over the coming decades.

## Data availability statement

The original contributions presented in the study are included in the article/supplementary material, further inquiries can be directed to the corresponding author.

## Author contributions

DT contributed significantly to framework, performed the data analyzes and wrote the part of methods and results. CM contributed significantly to analysis and wrote the part of introduction and discussion. QH contributed significantly to analysis and manuscript preparation. All authors contributed to the article and approved the submitted version.

## Funding

This research was supported by National Social Science Fund of China (21BRK008), the National Key R&D Program of China (2020YFC2003000) and fund for Building World-Class Universities (disciplines) of Renmin University of China (KYGJC2022008).

## Conflict of interest

The authors declare that the research was conducted in the absence of any commercial or financial relationships that could be construed as a potential conflict of interest.

## Publisher’s note

All claims expressed in this article are solely those of the authors and do not necessarily represent those of their affiliated organizations, or those of the publisher, the editors and the reviewers. Any product that may be evaluated in this article, or claim that may be made by its manufacturer, is not guaranteed or endorsed by the publisher.

## Footnotes

^1^Data and documentation are available at http://class.ruc.edu.cn

^2^The missing rate is high and mainly caused by income. The excluded cases were more likely to be widowed, live in rural area, less educated, and with poor health status. The excluded cases scored higher in depressive symptoms and lower in life satisfaction. However, there no significant differences were found regarding age, family ties, and friendship ties between the two groups. When we tried to delete the variable of income in the models, most results did not change.

^3^Functional limitations were measured by a scale that included 15 items of activities of daily living, which included giving someone a call, dressing oneself, taking a shower; having a meal, using toilet, moving to a chair from bed, walking indoor, walking outside, going up and down, taking public transportation, shopping, managing money, cooking, doing housework, lifting something weighing 5 kg. The participants were asked if they could finish the 15 items by themselves. They could choose an answer from three options: "without difficulty," "needing some help," "I cannot do it at all," coded as 1, 2, 3, respectively The Cronbach’s alpha was 0.885.

^4^The list of chronic diseases consisted of 24 common chronic diseases, including hypertension, heart diseases, diabetes, cerebrovascular diseases, kidney diseases, liver diseases, tuberculosis, rheumatoid, neck/lumbar diseases, arthritis, breast diseases, reproductive system diseases, prostate-related diseases, urology diseases, glaucoma\cataract, cancer, dementia, osteoporosis, respiratory diseases, nervous system diseases, digestive system diseases, Parkinson’s disease, deafness, other chronic diseases.
